# Transcriptomic Signature-Guided Depletion of Intermediate Alveolar Epithelial Cells Ameliorates Pulmonary Fibrosis in Mice

**DOI:** 10.21203/rs.3.rs-6623390/v1

**Published:** 2025-05-21

**Authors:** Yong Zhou, Fei Peng, Chun-sun jiang, Zhen Zheng, Shahram Aliyari, Dan Shan, Aaryan Sabharwal, Qinyan Yin, Chao He, Joseph Lasky, Victor Thannickal

## Abstract

Single-cell RNA sequencing (scRNA-seq) analyses of human and murine models of pulmonary fibrosis have uncovered multiple novel cell states involved in lung repair and regeneration, including an intermediate epithelial cell state identified as the KRT5-/KRT17+ aberrant basaloid cells in humans and the cytokeratin 8-positive alveolar differentiation intermediate (Krt8+ ADI) in mice. However, the specific contributions of these transitional cells to fibrogenesis remain poorly understood. In this study, we introduce a novel RNA-sensing-dependent protein translation technology that enables selective targeting and functional interrogation of Krt8+ ADI cells both *in vitro* and *in vivo* . Through transcriptomic analysis, we identified small proline-rich protein 1A ( *SPRR1A* ) mRNA as a highly specific marker shared by mouse Krt8+ ADI cells and human KRT5-/KRT17+ aberrant basaloid cells, effectively distinguishing them from other lung cell populations during lung injury and repair. Leveraging this transcriptomic specificity, we deployed programmable RNA sensors to drive EGFP reporter expression selectively in Krt8+ ADI cells *in vivo* . EGFP-labeled cells isolated from mouse lungs closely recapitulated the transcriptomic and phenotypic features of Krt8+ ADI cells, validating efficient and specific targeting. To assess the functional role of these cells, we established an RNA-sensing-dependent diphtheria toxin receptor (DTR) system to selectively ablate Sprr1a-expressing cells following diphtheria toxin (DT) administration. Targeted depletion of *Sprr1a* + cells significantly reduced fibrosis in bleomycin-injured mice, underscoring the pathogenic role of these transitional epithelial cells in lung fibrogenesis. Collectively, our findings define intermediate alveolar epithelial cells as key drivers of fibrosis and suggest that targeting this population may offer new therapeutic avenues for pulmonary fibrosis.

Transcriptomic signature, transitional AT2, lung fibrosis

### INTRODUCTION

Pulmonary fibrosis is a debilitating condition characterized by the progressive scarring of lung tissue. Central to the pathology of this disease is the failed regeneration and improper repair of lung tissue, processes that are not yet fully understood (1-3). Under normal conditions, the lung has the capacity to regenerate and repair following injury, primarily through the proliferation and differentiation of resident stem cells (4). This regenerative process is tightly regulated in healthy individuals, efficiently restoring lung architecture and function. However, in pulmonary fibrosis, this regenerative process is disrupted, often leading to aberrant wound healing where excessive connective tissue builds up, severely compromising respiratory function (5). The failure of lung regeneration in fibrotic conditions can be attributed to several interconnected factors. These include altered stem cell functionality due to cellular senescence and/or aging, genetic predisposition, changes in the local microenvironment/stem cell niche, and an imbalance in cytokine and growth factor signaling which contribute to the persistence of transitional cell states (6, 7). The persistence of such transitional cells may lead to a vicious cycle of damage and ineffective repair, culminating in non-resolving fibrosis.

Recent advancements in single-cell RNA sequencing (scRNA-seq) technologies have greatly enhanced our understanding of cellular behavior during lung injury and repair. scRNA-seq enables the detailed analysis of gene expression profiles at the single-cell level, allowing for the detection of rare cell populations, including intermediate cell states. In the context of lung repair, these intermediate states may represent pivotal points in the differentiation path from one cell type to another (8-10). Alternatively, they may signal aberrant differentiation patterns in diseased conditions (9, 11, 12).

Surfactant-producing cuboidal alveolar type II (AT2) cells function as a facultative progenitor for the alveolar epithelium. Following injury, AT2 cells can proliferate and differentiate into alveolar type I (AT1) cells, thereby replenishing the alveolar epithelium and maintaining proper lung function. However, disruptions in these processes can impede regeneration and contribute to pathological states such as pulmonary fibrosis (13, 14). Single-cell analyses of both human and murine models of pulmonary fibrosis have uncovered multiple novel cell states involved in the recovery of the alveolar epithelium following injury. For instance, AT2 cells initially proliferate post-injury, then exit the cell cycle and enter a transitional state before differentiating into AT1 cells. This transitional state is marked by cell cycle cessation, reduced expression of AT2 markers, a cuboidal or semi-spread morphology, and expression of distinct signature genes such as cytokeratin 8 (*Krt8*).

A transitional state known as the Krt8+ alveolar differentiation intermediate (Krt8+ ADI), has been observed post-bleomycin injury, peaking during the fibrotic phase around days 10 to 14, and subsiding once the injury resolves (8, 15). In a separate study, Kobayashi and colleagues reported a Cldn4+Krt19+ pre-alveolar type 1 transitional cell state (PATS), which is involved in the differentiation trajectory of AT2 to AT1 cells in both organoid and lung injury models (10). Similarly, Choi et al. identified a Krt8+Cldn4+ progenitor intermediate state, termed damage-associated transient progenitors (DATP), that emerges following bleomycin-induced lung injury (9). The transitional AT2 cells have also been observed in other studies, including loss of *Cdc42* during pneumonectomy (16), *Sin3a*-induced senescence (17), and *NOTCH* signaling activation during *Pseudomonas aeruginosa* infection (18). Moreover, transcriptional analyses have revealed that murine intermediate progenitors share similarities with KRT5-/KRT17+ cells and aberrant basaloid cells identified in human idiopathic pulmonary fibrosis (IPF) (8-10). A recent study also demonstrated that human AT2 cells transdifferentiate into metaplastic alveolar-basal intermediate (ABI) cells in response to mesenchymal fibrotic signaling (19).

While pulmonary fibrosis is characterized by the accumulation of transitional AT2 cells with impaired AT1 differentiation, the precise role of these transitional cells in the pathogenesis of fibrosis remains unclear. Previous studies have shown that AT2-specific p53 activation *via Mdm2* deletion, and pneumonectomy followed by AT1 ablation, lead to an accumulation of PATS, correlating with extensive pulmonary fibrosis in mice (20). In contrast, simultaneous ablation of PATS and transitional alveolar fibroblasts in Ager-diphtheria toxin receptor (DTR) mice resulted in lung tissue simplification and emphysema development. Furthermore, studies on *Krt8* knockout mice revealed protection against bleomycin-induced pulmonary fibrosis. Detailed analyses showed that in *Krt8*$^{+/+}$ mice, there was significant retention of transitional AT2 cells with incomplete AT1 regeneration by Day 21 post-bleomycin administration, whereas in *Krt8*$^{-/-}$ mice, transitional AT2 cells had largely resolved, with substantial restoration of AT1 cells (21). While these findings suggest a critical link between the transitional AT2 cell state and lung fibrosis development, it is not known if direct targeting of transitional cells post-fibrosis induction is capable of promoting regeneration and mitigating fibrosis.

In this study, we employed a novel RNA-sensing-dependent protein translation technology, known as CellREADR (Cell access through RNA sensing by Endogenous ADAR) (22), to detect small proline-rich protein 1A (*SPRR1A*), a unique transcriptomic marker of mouse Krt8+ ADI cells and human KRT5/KRT17+ aberrant basaloid cells, and to activate the translation of effector proteins, such as enhanced green fluorescent protein (EGFP) and diphtheria toxin receptor (DTR), in transitional AT2 cells. We demonstrate the efficacy of this approach by labeling Krt8+ ADI cells with EGFP, enabling the isolation of transitional AT2 cells from fibrotic mouse lungs. Our findings show that *Sprr1a*-guided depletion of Krt8+ ADI cells attenuates bleomycin-induced pulmonary fibrosis in mice. This transcriptomic signature-guided cell-targeting technology provides a valuable tool for investigating the functional roles of specific cell populations identified by scRNA-seq, both *in vitro* and *in vivo*.

### RESULTS

#### Validation of *in vivo* RNA-sensing-mediated translation of EGFP reporter in mouse alveolar epithelial cells

The RNA-sensing-dependent protein translation technology harnesses the activity of adenosine deaminases acting on RNA (ADAR), ubiquitously expressed in mammalian cells (23), to detect specific cellular RNAs and activate translation of effector proteins selectively within target cells (22, 24, 25) (**Figure 1A**). To evaluate the effectiveness of this technology for targeted gene expression within alveolar epithelial cells of mouse lungs, we designed two sensor RNAs (sesRNAs) that specifically recognize *Sftpc* mRNA in AT2 cells and *Pdpn* mRNA in AT1 cells through sequence-specific base pairing. Each sesRNA incorporates an ADAR-editable stop codon (UAG) that functions as a translational switch. Upon hybridization with target mRNA, ADAR-mediated adenosine-to-inosine editing alters the UAG stop codon to a UIG tryptophan codon, thus initiating translation of an effector RNA (efRNA) encoding EGFP. The resulting translation produces a fusion protein with an N-terminal peptide and C-terminal EGFP, subsequently separated by self-cleavage at the T2A site, releasing the EGFP (**Figure 1A**).

Prior to *in vivo* gene delivery, we validated the specificity of *Sftpc*-sesRNA- and *Pdpn* sesRNA-mediated *EGFP* efRNA expression *in vitro* using HEK293 cells. Cells were co-transfected with plasmids encoding full-length *Sftpc* or *Pdpn* cDNAs fused to mCherry or mCherry alone, together with their corresponding sesRNAs. *EGFP* expression was observed only in cells co-transfected with either pSftpc-mCherry (**Figure 1B**, left panel) or pPdpn-mCherry (**Figure 1C**, left panel) plasmids, but not in those receiving a control pmCherry plasmid alone.

We next tested *in vivo* expression using adeno-associated virus variant 6.2FF (AAV6.2FF), which efficiently transduces lung epithelial cells (26-29), to deliver sesRNA-*EGFP* expression cassettes into normal *Sftpc-Cre;Rosa-tdT* mice. Notably, *Sftpc*-sesRNA-guided EGFP expression was restricted to tdTomato-labeled Sftpc+ AT2 cells (**Figure 1B**, right panel, yellow signal). Regions lacking EGFP signal (red fluorescence) represent untransduced areas and served as internal controls, highlighting the specificity of expression. In contrast, *Pdpn*-sesRNA did not induce EGFP expression in tdT-labeled AT2 cells but instead labeled large, thin, flat cells with extended cytoplasmic processes that stretch over the alveolar surface, morphologically consistent with AT1 cells (**Figure 1C**, right panel). These findings demonstrate the cell-type-specific expression capability of our RNA-sensing system in alveolar epithelial cells *in vivo*.

**Identification of *SPRR1A* as a distinct transcriptomic signature of Krt8+ ADI cells in mice and KRT5-/KRT17+ aberrant basaloid cells in human IPF**

Alveolar epithelial cells in transitional states express markers of both AT2 cells and AT1 cells (8-10). A distinct subset of these transitional cells, termed Krt8+ ADI cells, emerges following bleomycin-induced injury in mice, peaking during the fibrotic phase (8, 15). However, analysis of a publicly available scRNA-seq dataset (GSE141259) reveals that *Krt8* expression is not confined to Krt8+ ADI cells but is distributed across various lung cell populations (**Figure 2A**).

In contrast, we identified *Sprr1a* as a transcript specifically enriched in Krt8+ ADI cells, exhibiting significantly higher expression in this population relative to other lung cell types (**Figure 2A**). To verify these bioinformatics observations, we performed RNA fluorescence in situ hybridization (FISH) combined with immunofluorescence to co-localize *Sprr1a* mRNA with Krt8 and Sftpc proteins in lung tissues from bleomycin-treated and control C57BL/6 mice. In fibrotic lungs, *Sprr1a* mRNA was detected primarily in $Krt8^{+}Sftpc^{+}$ cells, and more robustly in $Krt8^{high}Sftpc^{low}$ cells located within fibrotic regions (**Figure 2B**). Notably, not all Krt8+ cells expressed *Sprr1a*, confirming the specificity of *Sprr1a* for a subset of these cells, distinct from the broader Krt8 expression observed in single-cell transcriptomic analysis (GSE141259). Furthermore, cells with higher Sftpc levels lacked *Sprr1a* expression, consistent with a more undifferentiated state of AT2 cells and supporting the concept that transitional AT2 cells represent an intermediate state of AT2-AT1 differentiation. In contrast, control lungs exhibited minimal *Sprr1a* expression and little to no Krt8 expression in alveolar regions, reinforcing the injury-specific induction of these markers (**Figure 2B**).

Analysis of independent scRNA-seq datasets from the IPF Cell Atlas revealed selective enrichment of *SPRR1A* mRNA in KRT5-/KRT17+ aberrant basaloid cells in human IPF lungs (**Supplemental Figure 1**). To validate these transcriptomic findings, we performed immunofluorescence staining on lung tissues from both patients with IPF and healthy control subjects. In IPF lungs, SPRR1A signals were primarily localized to KRT17+/KRT5- epithelial cells lining fibroblastic foci and honeycomb cysts (**Figure 2C**). In contrast, SPRR1A signals were absent in healthy lungs. Distinct KRT5+ signals and lower levels of KRT17 expression were observed in the normal airway epithelium, with minimal overlap between the two markers (**Figure 2C**). Independent KRT5+ signals were also detected in the airway epithelium of IPF lungs. Collectively, these bioinformatic and experimental analyses establish *SPRR1A* as a robust and specific transcriptomic marker for both mouse Krt8+ ADI cells and human KRT5-/KRT17+ aberrant basaloid cells. While *Krt8* is broadly expressed across various transitional cell states, its lack of specificity limits its use as a unique identifier, unlike *SPRR1A*, which identifies a unique subpopulation of injury-associated epithelial cells during lung repair and fibrosis.

**Targeted *in vivo* labeling of Krt8+ ADI cells using *Sprr1a*-guided RNA-sensing technology**

Having identified *Sprr1a* as a specific transcriptomic marker of Krt8+ ADI cells, we next applied cutting-edge programmable RNA-sensing technology to selectively target these transitional AT2 cells *in vivo*. We designed a 102-nucleotide sesRNA to specifically bind exon 2 of *Sprr1a* mRNA. The specificity and efficiency of this *Sprr1a* sesRNA in driving expression of an EGFP reporter were first validated *in vitro* using HEK 293 cells engineered to express *Sprr1a* (**Figure 3A**). To evaluate whether the *Sprr1a* sesRNA could selectively direct reporter expression to Krt8+ ADI cells *in vivo*, we delivered AAV6.2FF vectors encoding the *Sprr1a* sesRNA-*EGFP* reporter construct into *Krt8-Cre;Rosa-tdT* mice subjected to bleomycin injury, as well as into saline-treated control mice. In control lungs, Krt8 expression was restricted primarily to the airway epithelium, and no EGFP reporter signal was detected (**Figure 3B**). In contrast, bleomycin-treated lungs exhibited substantial colocalization of Krt8 (tdT+) and EGFP signals within alveolar regions across areas of both mild and severe fibrosis (**Figure 3C**), suggesting successful and specific targeting of Krt8+ ADI cells by the *Sprr1a* sesRNA system.

**Comparison of transcriptomic profiles of *Sprr1a*+ cells and Krt8+ ADI cells**

To determine whether the EGFP-labeled *Sprr1a*+ cell population corresponds to Krt8+ ADI cells in the fibrotic mouse lung, we performed scRNA-seq on whole lung cells isolated from single-dose bleomycin-treated C57BL/6 mice transduced with an AAV6.2FF vector encoding *Sprr1a* sesRNA-*EGFP*. Expression analysis of epithelial structural genes (*Krt8*, *Krt18*, *Krt7*, *Krt19*) and injury-associated markers previously linked to the $Krt8^{+}$ ADI state (*Sprr1a*, *Areg*, *Hbegf*, *Tnfrsf12a*, *Itgb6*, *Cldn18*, *Cldn4*, *Cryab*, *Cdkn1a*) revealed that EGFP+ cells exhibited significantly higher expression of most of these markers compared to EGFP- cells (**Figure 4A**). This suggests that *Sprr1a*-driven EGFP+ cells transcriptionally resemble the transitional Krt8+ ADI population.

To further quantify this relationship, we conducted correlation analyses based on the top 50 differentially expressed genes (DEGs) between EGFP+ and Krt8+ ADI cells. Strong correlation was observed using multiple similarity metrics: Spearman (0.81), cosine similarity (0.76), and Pearson (0.64) (**Figure 4B**), indicating robust transcriptional convergence between the two populations.

UMAP visualization of single-cell transcriptomes revealed that EGFP+ cells largely co-localized with Krt8+ ADI cells, although at a lower density, likely reflecting suboptimal viral transduction efficiency and/or limited EGFP transcript capture during scRNA-seq. These EGFP+ cells were primarily enriched within specific Leiden clusters, further supporting that the sesRNA-based *Sprr1a*-EGFP system selectively labels Krt8+ ADI-like transitional epithelial cells (**Figure 4C**).

Together, these findings demonstrate that *Sprr1a* serves as a reliable transcriptomic marker of Krt8+ ADI cells and that RNA-sensing-based EGFP labeling faithfully captures this transitional epithelial population in the injured lung.

**Phenotypic characterization of *Sprr1a*+ cells isolated from mouse lungs**

Transitional AT2 cells are known to exhibit features of cellular senescence (8, 10, 21, 30). To assess whether *Sprr1a*+ cells exhibit a senescence-like phenotype, we purified EGFP-labeled *Sprr1a*+ cells from bleomycin-treated mouse lungs using flow cytometry and subjected the freshly isolated cells to senescence analysis via cytospin preparation. For comparison, *Sftpc*+ AT2 cells were isolated from saline-treated control lungs using an AAV6.2FF vector encoding *Sftpc* sesRNA-*EGFP*. *Sprr1a*+ cells demonstrated a markedly higher degree of cellular senescence than *Sftpc*+ AT2 cells, as evidenced by both broader distribution and stronger expression of senescence markers, including senescence-associated β-galactosidase (SA-β-gal), p21 (Cdkn1a), and p53 (Tp53) in *Sprr1a*+ cells vs. *Sftpc*+ AT2 cells (**Figure 5A**, **5C**, and **5D**). Interestingly, expression of p16 (Cdkn2a), another senescence-associated gene, remained low or undetectable in both *Sprr1a*+ and *Sftpc*+ AT2 cells (**Figure 5B**), suggesting a p16-independent senescence-like phenotype.

To further characterize the regenerative potential of *Sprr1a*+ cells, we assessed their ability to form alveolar organoids in 3D Matrigel cultures. While *Sprr1a*+ cells exhibited reduced colony-forming efficiency compared to *Sftpc*+ AT2 cells from healthy lungs, the colonies that did form were significantly larger in size (**Figure 5E**). Interestingly, Ki67 immunostaining identified a small subset of *Sprr1a*+ cells (~1%) displaying high proliferative activity *ex vivo* (**Figure 5F**).

Collectively, these findings confirm a senescence-like phenotype of *Sprr1a*+ cells with a smaller subset that evades cell cycle arrest and assumes a hyper-proliferative state.

#### *Sprr1a*-guided depletion of Krt8+ ADI cells attenuates pulmonary fibrosis in mice

To investigate the role of transitional AT2 cells in lung fibrogenesis, we directly targeted Krt8+ ADI cells using *Sprr1a* mRNA-sensing-dependent protein translation coupled with the DT/DTR system. Krt8+ ADI transitional cells initially appeared on day 2 post-bleomycin lung injury, peaked around days 10–14, and persist until day 36 (8). Our strategy focused on diminishing the population of these cells at their peak. On day 8 post-bleomycin treatment, we administered AAV6.2FF-*Sprr1a* sesRNA-*DTR* vectors to C57BL6 mice *via* oropharygneal aspiration, followed by DT administration on day 14 (**Figure 6A**). This regimen also minimized the potential for viral infection to impact bleomycin-induced lung injury and inflammation. Integrated staining for *Sprr1a* mRNA *via* RNA FISH, DTR expression by immunofluorescence, and cell death by TUNEL revealed that the DTR was exclusively expressed in *Sprr1a*+ cells within fibrotic lung tissues (**Figure 6B**, upper panel). To quantify *Sprr1a*+ cell death, we infected bleomycin-treated mice with AAV6.2FF-*Sprr1a* sesRNA-*ZsG-DTR* viruses, in which DTR-expressing *Sprr1a*+ cells were labeled by ZsGreen1. Flow cytometry analysis of ZsGreen1-labeled *Sprr1a*+ cells demonstrated that DT treatment resulted in 29.8 ± 1.9% early apoptosis, 5.3 ± 0.6% late apoptosis, and 3.0 ± 0.3% necrosis. In contrast, untreated mice showed 10.9 ± 1.1% early apoptosis, 0.9 ± 0.3% late apoptosis, and 1.3 ± 0.2% necrosis (**Figure 6C**). These results demonstrate the effectiveness of integrating *Sprr1a* mRNA-sensing-dependent DTR expression with DT treatment to deplete transitional AT2 cells in fibrotic mouse lungs. Subsequent analysis of lung fibrosis showed that this depletion significantly mitigated pulmonary fibrosis, as evidenced by Masson's trichrome, which highlighted reduced collagen deposition in the lungs (**Figure 6D**), decreased total hydroxyproline content (**Figure 6E**), and lowered expression of fibrotic markers such as collagen I, fibronectin, and α smooth muscle actin (**Figure 6F**). A notable observation was that approximately 11.8% of *Sprr1a*+ cells were apoptotic in mice that did not receive DT treatment by day 15 post-bleomycin exposure (**Figures 6B**, lower panel and **6C**). This finding suggests that transitional AT2 cells may naturally undergo turnover during the course of lung injury and repair. To further characterize the dynamics of *Sprr1a*$^{+}$ cell apoptosis, we analyzed ZsGreen1-labeled *Sprr1a*+ cells at multiple time points (days 12, 15, 17, 19, 24, and 29) following bleomycin administration (day 0) and viral infection (day 8). Apoptosis rates gradually increased from 5.9 ± 2.4% at day 12, peaked between days 17 and 19 (24.0 ± 1.0%  to 24.6% ± 4.6%), and subsequently declined, reaching 10.9 ± 2.5% by day 29 (**Supplemental Figure 2**).  These results indicate that transitional AT2 cells exhibit a dynamic pattern of apoptosis during lung fibrosis progression and resolution, highlighting their transient nature in the injured lung microenvironment.

Collectively, our study demonstrates that targeted depletion of Krt8+ ADI transitional cells using a distinct transcriptomic signature effectively attenuates pulmonary fibrosis in mice. Additionally, our findings suggest that apoptosis of these cells, in addition to their terminal differentiation to AT1 cells, may serve as an intrinsic mechanism facilitating the turnover of transitional AT2 cells during the repair and regeneration of injured lungs.

### DISCUSSION

Recent advancements in scRNA-seq technologies have considerably enhanced our understanding of cellular dynamics during lung injury and repair processes. However, beyond transcriptome profiling, it is critical to actively monitor and manipulate specific cell types associated with disease to further clarify their roles in the pathogenesis of pulmonary fibrosis. In this study, we introduced a novel *in vivo* cell-targeting strategy that utilizes distinct transcriptomic signatures sourced from publicly available RNA-seq datasets along with CellREADR technology. Our approach successfully labeled Krt8+ ADI transitional AT2 cells, enabling their isolation during lung injury and repair in mice. Notably, targeted depletion of Krt8+ ADI cells resulted in attenuated pulmonary fibrosis in mice. This provides the first direct evidence of the critical role of transitional AT2 cells in lung fibrogenesis by specifically targeting this cellular population. Compared to traditional lineage-tracing methods in transgenic animal models, our *in vivo* transcriptomic signature-guided cell targeting approach offers a rapid, more cost-effective, and highly specific method for studying the function of distinct cell lineages, particularly in the context of disease progression. A major finding of this study is the identification of *SPRR1A* as a robust and specific transcriptomic marker for both mouse Krt8+ ADI cells and human KRT5-/KRT17+ aberrant basaloid cells. The identification of *SPRR1A* as a shared marker across species strengthens the translational relevance of our findings and provides a new avenue for targeted intervention strategies in pulmonary fibrosis.

*In vivo Sprr1a*-guided labeling of transitional AT2 cells with a fluorescent protein allowed us to purify these cells from mouse lungs for further *in vitro* analyses. SA-β-gal staining of uncultured *Sprr1a*+ cells revealed that the majority exhibit a senescent phenotype, as previously suggested for the Krt8+ ADI subpopulation (8, 10, 30). Interestingly, these cells did not express p16/Cdkn2a, a marker strongly associated with irreversible senescence (31, 32). Recent studies suggest two subsets within both murine and human transitional AT2 cells: one undergoing a transient senescence-like state potentially leading to differentiation into AT1 cells, and another that is permanently senescent and fails to differentiate (21, 33). In a single bleomycin-induced mouse lung fibrosis model, senescence in transitional AT2 is transient, occurring as these proliferating cells exit the cell cycle in preparation for AT1 differentiation. Conversely, permanently arrested transitional AT2 cells were infrequent in this model (34). However, under the conditions of repeated bleomycin exposure, which models chronic lung fibrosis, transitional AT2 cells enter an aberrant basaloid state characterized by permanent senescence, inability to differentiate into AT1 cells, and contribution to sustained or progressive fibrosis (21). Examination of transitional cell states in human IPF revealed similar cellular phenotypes: transient senescence in "ABI1" or "AT2 transitional" cells and permanent arrest in "ABI2," "KRT5-/KRT17+," or "aberrant basaloid" cells, mirroring the permanently arrested transitional AT2 cells in mice (11, 33, 35). Our analysis of transitional AT2 cells directly isolated from mice treated with a single dose of bleomycin showed general markers of senescence but lacked p16/Cdkn2a expression, supporting the hypothesis that these cells are in a transiently senescent state. This distinction between transient and permanent senescence in transitional AT2 cells enhances our understanding of their roles in lung injury and repair processes.

To investigate the stem/progenitor cell properties of transitional AT2 cells, we cultured *Sprr1a*+ cells isolated from fibrotic mouse lungs in Matrigel to assess their ability to form cell colonies. Our results revealed that *Sprr1a*+ cells formed significantly fewer colonies in 3D organoid culture compared to AT2 cells isolated from normal mouse lungs. This reduction in colony formation aligns with our observation that *Sprr1a*+ cells possess a senescence-like phenotype, impairing their ability to proliferate robustly. Interestingly, while the number of cell colonies formed by *Sprr1a*+ cells in Matrigel was markedly reduced, the colonies that did emerge were significantly larger than those formed by normal AT2 cells. This observation suggests that a subset of the *Sprr1a*+ population may exhibit a higher proliferative rate once the cell cycle arrest is bypassed or mitigated, hinting at heterogeneity within the transitional AT2 cell population. While the assertion that a subset of transitional AT2 cells have high proliferative potential remains speculative, the concept is supported indirectly by literature identifying similar characteristics in cells involved in lung repair mechanisms (36-39). A recent study suggests that hyperplastic cells historically designated as AT2 cells in diffuse alveolar damage are in fact transitional AT2 cells (36). Furthermore, emerging evidence suggests that the hyperplastic epithelial cells lining the fibroblastic foci in IPF are analogous to mouse transitional AT2 cells (37-39). These fibroblastic foci represent areas of active fibrotic remodeling and are thought to be critical in disease progression. The larger colony size observed in *Sprr1a*+ cells could result from selective pressures favoring a more proliferative subset capable of expanding rapidly despite an overall reduction in colony-forming efficiency. Our study adds to the growing body of evidence suggesting a dynamic role of transitional AT2 cells in lung fibrosis. It emphasizes the need for further investigation into the specific functions and characteristics of these cells, including their potential heterogeneity, proliferative capacity, and role in disease progression and resolution.

In this study, *Sprr1a*-guided DTR expression and subsequent DT administration successfully diminished transitional AT2 cell populations by approximately 38% at their peak in fibrotic mouse lungs. The targeted depletion of these cells attenuated bleomycin-induced pulmonary fibrosis, suggesting a “causal” role for transitional AT2 cells in lung fibrogenesis. To our knowledge, this is the first report to demonstrate the essentiality of transitional AT2 cells in fibrosis of the lung. Intriguingly, we observed that around 11.8% of *Sprr1a*+ cells in the control group (untreated with DT) also underwent apoptosis at day 15 post-bleomycin administration, coinciding with the peak abundance of transitional AT2 cells. This observation suggests a natural turnover of these cells even in the absence of DT-mediated cell death. Further longitudinal analysis of apoptosis in *Sprr1a*+ cells reveals a dynamic pattern: apoptosis rates gradually increased from day 12, peaked between days 17 and 19, and then declined by day 29. These results indicate that transitional AT2 cells are not a stable population but instead undergo programmed cell death as part of the normal progression and resolution of lung fibrosis. This dynamic apoptotic behavior highlights the transient nature of transitional AT2 cells within the injured lung microenvironment and suggests that tightly regulated cell turnover may be critical for successful epithelial regeneration and restoration of alveolar homeostasis. The phenomenon of natural clearance of excess transitional AT2 cells in the context of resolving bleomycin-induced lung fibrosis aligns with our results demonstrating that targeted depletion of these cells at their peak ameliorates fibrosis in this model. Corroborating our findings, analysis of single cells to identify enrichment of gene programs demonstrated that Krt8+ ADI transitional cells exhibited elevated expression levels of genes that promote cell death compared to other epithelial cells (8). Furthermore, a recent study has localized transitional AT2 cells not only within the fibrotic lung parenchyma but also within the airspaces, indicative of cell death and sloughing processes (34). Additional studies have shown that transitional AT2 cells located at fibroblastic foci exhibit apoptotic features (40, 41).

Together, our findings highlight the importance of transitional AT2 cells in the fibrotic process and underscore the therapeutic potential of targeting these cells to modulate fibrosis. Our data also suggest that apoptosis may serve as a natural regulatory mechanism to prevent the prolonged presence of transitional AT2 cells, which, if left unchecked, could contribute to pathological fibrotic remodeling. Further studies are warranted to elucidate the precise molecular pathways involved in the regulation of transitional AT2 cells. Such insights are crucial for designing interventions that could either enhance the natural resolution mechanisms or prevent the excessive accumulation of these cells, potentially offering new therapeutic avenues for treating fibrotic lung diseases.

### METHODS

#### sesRNA design

Sensor sequences were designed based on the criteria described by Qian et al. (22). Briefly, sesRNAs were constructed as antisense sequences complementary to the mRNAs of *Sftpc*, *Pdpn*, and *Sprr1a* mRNAs, with an optimal length of 200–300 nucleotides. Each sesRNA was linked in-frame to an effector RNA (*EGFP*, *ZsGreen1*, or *DTR*), forming a single open reading frame. A TAG stop codons was introduced near the midpoint of the sesRNA sequence (typically 80-220 nucleotides from the 5' end) to regulate translation. To create a strategic A–C mismatch, 5'-TGG-3' sequences in the sesRNA that would pair with 5'-CCA-3' in the target mRNA were mutated to 5'-TAG-3'. Additionally, to avoid unintended translation initiation, no ATG start codons were included downstream of the central TAG stop codon. The sesRNA cDNAs were synthesized as gBlocks (Integrated DNA Technologies) and subcloned into an AAV vector backbone. All sesRNA sequences used in this study are listed in **Supplementary Table 1**.

#### AAV virus production and purification

HEK293T cells were maintained in a proliferative state by passaging at a 1:2 ratio for at least two consecutive days. For AAV production, cells were seeded into 10-cm culture dishes at a density of 4–8 × $10^5$ cells/mL in antibiotic-free DMEM to achieve ~70–80% confluency the following day. Cells were co-transfected with a pAAV2-based expression vector (e.g., pAAV2-CAG-mTagBFP-Ses-P2A-GFP) and the helper plasmid pDGM6.2FF at a 1:3 ratio using PEI (1 mg/mL) in Opti-MEM. Specifically, 5 µg of expression vector and 15 µg of pDGM6.2FF were diluted in 7.5 ml Opti-MEM, while 900 µl of PEI was diluted in a separate 7.5 ml Opti-MEM. The PEI solution was added to the DNA mixture, incubated at room temperature for 10–15 minutes to allow complex formation, and then added dropwise to the cells. After 5 hours, the medium was replaced with fresh DMEM containing 10% FBS. Transfection efficiency was assessed by BFP expression at 24 hours. Cultures with ≥80% BFP-positive cells were maintained for a total of 72 hours post-transfection. Cells and media were harvested by centrifugation at 500 × g for 10 minutes and stored at –80 °C overnight.

For virus extraction, cell pellets were resuspended in 47.5 ml of PBS containing protease inhibitors and split into two 50 ml tubes. Sodium deoxycholate was added to a final concentration of 0.5%, along with benzonase (50 U/mL), and the lysates were incubated at 37 °C for 1 hour. Following lysis, samples were centrifuged at 3,000 × g for 15 minutes to remove debris. The resulting supernatants, including those from the culture media, were combined, frozen overnight, and then thawed and incubated again at 37 °C for 1 hour. The viral lysate was clarified by a second centrifugation (3,000 × g, 15 min), and loaded onto a pre-equilibrated HiTrap heparin affinity column using a peristaltic pump at a rate of 1 ml/min. The column was washed sequentially with 50 ml PBS and 50 ml 200 mM NaCl in PBS. Virus was eluted in a stepwise manner using 1.5 ml of 300 mM, 3 ml of 350 mM, and 1.5 ml of 450 mM NaCl in PBS. Eluted fractions were filtered through a 0.2 µm syringe filter and concentrated using Amicon Ultra-4 centrifugal filters (100 kDa MWCO; Millipore) by centrifugation at 4,000 × g until a final volume of approximately 0.25 ml was reached. The concentrated virus was resuspended in

PBS to a final volume of 500 μl, aliquoted, and stored at –80 °C. Viral genome titers were quantified by RT-PCR.

**In situ hybridization**

In situ hybridization was performed using the miRCURY LNA miRNA ISH Optimization Kit (QIAGEN), following the manufacturer's One-Day miRNA ISH protocol optimized for formalin-fixed paraffin-embedded samples. Tissue sections (5 μm) were deparaffinized in xylene and rehydrated through a graded ethanol series. Sections were then treated with freshly prepared Proteinase K solution (diluted in Proteinase K buffer) for 10 minutes at 37 °C to permeabilize the tissue. Hybridization was carried out using double-digoxigenin-labeled LNA miRNA probes at a final concentration of 40 nM, diluted in formamide-free ISH buffer. Probes were denatured at 90 °C for 4 minutes prior to application. Hybridization was performed at 60 °C for 1 hour in a humidified hybridization chamber. Following hybridization, slides were subjected to a series of stringent washes with saline-sodium citrate buffers at the hybridization temperature to remove nonspecifically bound probes.

**Single-cell RNA-seq library preparation and analysis**

Single-cell RNA sequencing was performed using the 10x Genomics Fixed RNA Profiling (scRNA-seq Flex) chemistry. Viable lung cells were isolated from mice infected with AAV-*Sprr1a* SesRNA-*EGFP* and processed according to the manufacturer's protocol (10x Genomics Inc.). Briefly, single-cell suspensions were treated with a proprietary crosslinking reagent to preserve RNA integrity and stored at –80°C until all experimental groups were collected.

Target transcript capture and sample barcoding were performed using the Chromium Mouse Transcriptome Probe Set v1.0.1 (10x Genomics). Each sample was labeled with a unique sample-specific probe barcode. To enable detection of EGFP expression, a custom probe targeting EGFP was included during hybridization. After probe hybridization, samples were pooled and washed to remove unbound probes. Barcoded cells from pooled samples were encapsulated into Gel Bead-in-Emulsions (GEMs) using the Chromium Chip Q. Inside each GEM, nicked probe pairs were ligated, and unique molecular identifiers (UMIs), 10x GEM barcodes, and partial Read 1T sequences were incorporated through polymerase extension. Ligated and barcoded probe pairs were then amplified via sample index PCR to generate sequencing-ready libraries containing Illumina P5/P7 adapters and Read 2S sequences.

Library quality was assessed using the Agilent High Sensitivity DNA Kit on an Agilent 2100 Bioanalyzer, and library concentrations were measured with a Qubit 2.0 fluorometer. Libraries were pooled and sequenced at a final concentration of 750 pM using paired-end and dual-index reads on an Illumina NextSeq 2000 platform. Raw sequencing data were processed using Cell Ranger v9.0.0 (10x Genomics), and reads were aligned to the GRCm39-2020-A mouse reference genome using the Chromium Mouse Transcriptome Probe Set v1.1.1.

**Computational analysis of scRNA-seq datasets**

Single-cell RNA sequencing data from bleomycin-injured mouse lungs were processed using the Scanpy package (v1.9.0). Initial quality control involved filtering out cells with low gene counts or high mitochondrial transcript content. Data were normalized to a total count of 10,000 reads per cell and subsequently log-transformed. *EGFP*+ cells were identified based on non-zero expression of the *EGFP* gene. Dimensionality reduction was performed using principal component analysis (PCA), followed by construction of a neighborhood graph and clustering using the Leiden algorithm (resolution = 0.8). Two-dimensional embedding and visualization were conducted using Uniform Manifold Approximation and Projection (UMAP).

To define Krt8+ ADI-like cells, we curated a panel of marker genes based on Strunz et al (8). This panel included structural cytokeratins (*Krt8*, *Krt18*, *Krt7*, *Krt19*) and injury-response genes (*Sprr1a*, *Areg*, *Hbegf*, *Tnfrsf12a*, *Itgb6*, *Cldn18*, *Cldn4*, *Cryab*, *Cdkn1a*). Cells were annotated as Krt8+ ADI-like if they expressed the markers from this panel at a normalized expression level >1.

Differential gene expression analysis was performed using the rank-genes-groups function in Scanpy, applying the Wilcoxon rank-sum test. P-values were adjusted for multiple comparisons using the Benjamini–Hochberg procedure, and genes with an adjusted p-value < 0.05 were considered statistically significant. To evaluate transcriptomic similarity between EGFP+ and Krt8+ ADI-like populations, the top 50 differentially expressed genes (DEGs) from each group were selected. Pairwise similarity metrics, including Pearson correlation, Spearman correlation, and cosine similarity, were calculated using the SciPy library (v1.10). All plots, including violin plots, heatmaps, and UMAP visualizations, were generated using Seaborn (v0.12) and Matplotlib (v3.7).

**Organoid culture**

Single-cell suspensions were prepared from murine lungs through mechanical and enzymatic dissociation. tdTomato-labeled AT2 cells or EGFP+ epithelial cells were isolated by FACS into ice-cold FACS buffer containing HBSS, 25 mM HEPES, 2 mM EDTA, and 2% FBS. For each biological replicate, $1 \times 10^4$ sorted epithelial cells were combined with $5 \times 10^4$ Pdgfra+ primary lung fibroblasts in a 1:1 mixture of growth factor–reduced Matrigel (Corning) and MTEC plus medium. MTEC Plus consisted of DMEM/F12 (Invitrogen) supplemented with insulin/transferrin/selenium (Gibco), epidermal growth factor (Sigma), bovine pituitary extract (Sigma), and 5% FBS (Hyclone). The cell-Matrigel mixture was plated into Transwell inserts and allowed to polymerize at 37 °C for 1 hour. After polymerization, MTEC Plus medium was added to the lower chamber, and the culture medium was refreshed every other day. Organoid growth and morphology were monitored using a Nikon Eclipse Ts2-FL inverted fluorescence microscope.

**Animals and experimental protocol**

All animal procedures were approved by the Institutional Animal Care and Use Committees (IACUC) of Tulane University and University of Alabama at Birmingham. Twelve-week-old pathogen-free female mice were used for all experiments. Two transgenic mouse models were employed in this study: (1) *Krt8-Cre;Rosa-tdTomato* mice, generated by crossing *Tg(Krt8-cre/ERT2)17Blpn/J* mice with *B6.Cg-Gt(ROSA)26Sortm14(CAG-tdTomato)Hze/J* mice (The Jackson Laboratory, Bar Harbor, ME); and (2) *SFTPC-Cre;Rosa-tdTomato* mice, generated by crossing *B6.129S-Sftpc^tm1(cre/ERT2)Blh/J* mice with the same *Rosa*-tdTomato reporter line (The Jackson Laboratory). To induce tdTomato expression in Cre-expressing cells, tamoxifen was administered via intraperitoneal injection at a dose of 50 mg/kg per day for five consecutive days. Lung injury and fibrotic remodeling were induced by intratracheal administration of bleomycin sulfate (Almirall) dissolved in 50 μl sterile saline at a dose of 1.5 U/kg body weight. Control mice received an equal volume of saline.

For selective depletion of *Sprr1a*+ cells *in vivo*, mice underwent a sequential treatment protocol. On day 0, mice were anesthetized and administered intratracheal bleomycin. On day 8, an AAV vector encoding a sesRNA-guided *DTR* construct was delivered intratracheally for cell-specific targeting of *Sprr1a*+ cells. On day 14, DT was administered intratracheally to ablate DTR-expressing *Sprr1a*+ cells. Lung tissues were harvested on day 28 for fibrosis analyses.

At harvest, mice were euthanized, and lungs were processed for immunofluorescence, histology, or biochemical analyses. For immunofluorescence, lungs were excised and inflated with OCT compound before embedding. For histological analysis, lungs were fixed in 10% neutral-buffered formalin and paraffin-embedded. For biochemical analysis, fresh lung tissues were placed into pre-chilled tubes containing T-PER Tissue Protein Extraction Reagent (Thermo Fisher Scientific) supplemented with Complete Mini Protease Inhibitor Cocktail (Roche) at a ratio of 1 tablet per 10 ml of T-PER. Tissues were homogenized on ice and centrifuged at 9,000 × g for 10 minutes at 4 °C. Supernatants were collected, and total protein concentration was quantified using the BCA Protein Assay Kit (Thermo Fisher).

**Statistical analysis**

Statistical differences among treatment conditions were determined by one way analysis of variance (ANOVA) (Newman-Keuls method for multiple comparisons) or two-tailed Student's t test using GraphPad Prism 8 software. Values of $p < 0.05$ were considered significant.

**Data availability**

The single-cell RNA sequencing (scRNA-seq) data generated in this study will be deposited in the Gene Expression Omnibus (GEO). Accession numbers will be provided upon acceptance of the manuscript.

**Study approval**

The animal studies were conducted in accordance with the NIH guidelines for Care and Use of Laboratory Animals and approved by Institutional Animal Care and Use Committee at Tulane University and University of Alabama at Birmingham.

Additional detail on the methods is provided in the online Supplemental Materials.

### AUTHOR CONTRIBUTIONS

F.P., C.J., Z.Z., and Y.Z. designed the study; F.P., C.J., Z.Z., D.S., A.S., and Y.Q., and C.H. performed experiments; F.P., C.J., Z.Z., S.A., and Y.Z. analyzed data; C.H., J.A.L. and V.J.T. participated in discussion; F.P., J.S., Z.Z., and Y.Z. wrote the manuscript.

### ACKNOWLEDGEMENTS

The authors would like to thank Dr. Jay K. Kolls and Dr. Kejing Song for their assistance with scRNA-seq. This work was supported in part by NIH grants HL139584, HL156973, and HL174994.

### FIGURE LEGENDS

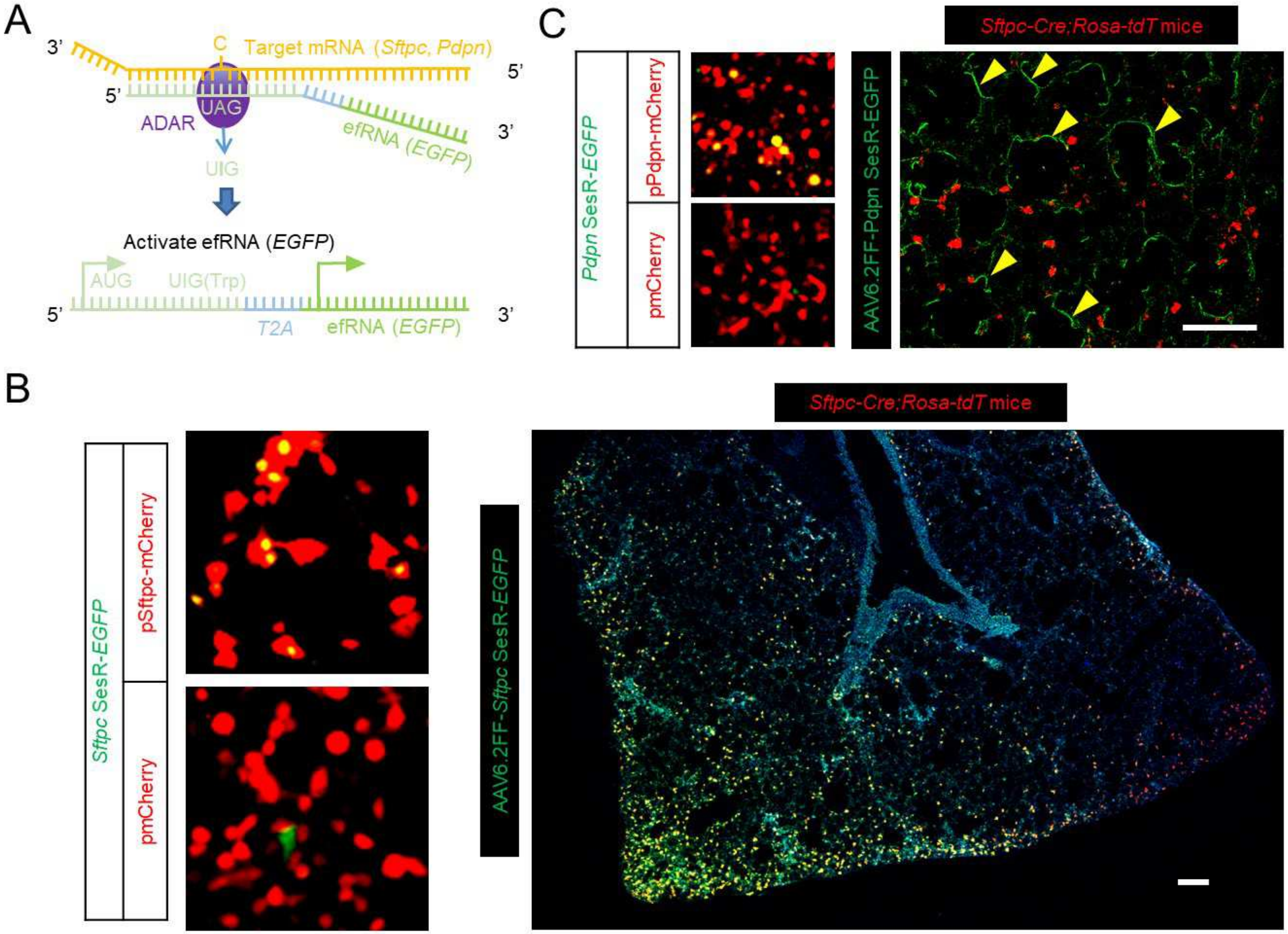

Figure 1: **Validation of *in vivo* RNA-sensing-mediated translation of EGFP reporter in mouse alveolar epithelial cells**. **A**, Schematic of RNA-sensing-based protein translation. The modular RNA consists of a 5' sensor region (sesRNA) and a 3' effector region (efRNA), separated by a T2A linker. The sesRNA is complementary to a target cellular RNA and contains a stop codon (UAG) that blocks translation to the efRNA. Upon hybridization with its target, ADAR enzymes are recruited to edit the UAG to a tryptophan codon (UGG), enabling translation of the downstream effector protein; **B**, *Left panel*: HEK 293 cells were co-transfected with *Sftpc* SesR-*EFGP* and either pSftpc-mCherry or control pmCherry. Confocal microscopy revealed EGFP expression in cells co-expressing Sftpc-mCherry (yellow signal), confirming Sftpc-dependent activation. *Right panel*: AAV6.2FF-*Sftpc* SesR-EGFP vectors were administered to *Sftpc-Cre;Rosa-tdT* mice. Confocal images of frozen lung sections showed co-localization of EGFP and tdT in the left lung lobe, indicating EGFP expression in AT2 cells. In contrast, the right lung lobe, particularly the distal tip, contained tdT+ AT2 cells that lacked EGFP expression, likely reflecting areas of incomplete viral transduction and thereby serving as internal negative controls; **C**, Similar experiments were conducted using *Pdpn*-targeting sesRNA to induce EGFP expression in *Pdpn*-expressing HEK 293 cells *in vitro* and AT1 cells *in vivo*. In *Sftpc-Cre;Rosa-tdTomato* mice, EGFP was detected in large, thin cells with extended cytoplasmic processes characteristic of AT1 morphology. Scale bar = 20 μm.

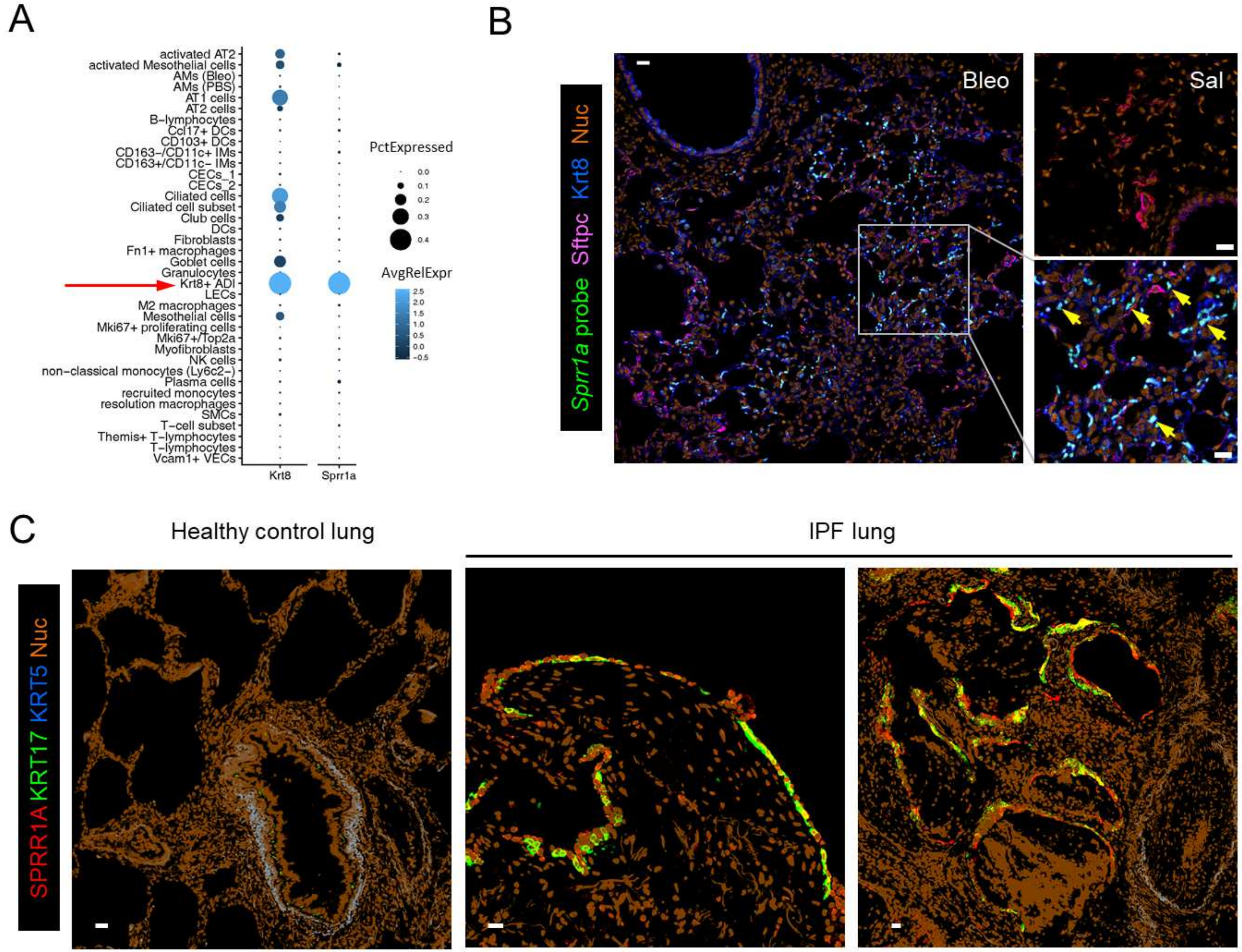

Figure 2: ***SPRR1A* is a distinct transcriptomic marker of Krt8+ ADI cells in mice and aberrant basaloid cells in human IPF**. **A**, Analysis of *Krt8* and *Sprr1a* expression across various major mouse lung cell populations using a publicly available scRNA-seq dataset (GSE141259); **B**, Co-localization of *Sprr1a* mRNA with Krt8 and Sftpc proteins in lung tissues from bleomycin (bleo)-treated and saline (sal)-treated C57BL/6 mice by RNA FISH combined with immunofluorescence staining. **C**, Co-localization of SPRR1A, KRT17, and KRT5 in lung tissues from patients with IPF and healthy control subjects by immunofluorescence staining. Scale bar = 20 μm.

Figure 3: **Targeted *in vivo* labeling of Krt8+ ADI cells via *Sprr1a* mRNA-guided EGFP expression**. **A**, HEK 293 cells were co-transfected with *Sprr1a* SesR-*H2BEFGP* and either pSprr1a-mCherry or the control vector pmCherry. Confocal imaging showed EGFP expression selectively in cells co-expressing Sprr1a-mCherry (yellow), confirming *Sprr1a*-specific activation of the RNA-sensing system. **B** and **C**, AAV6.2FF-*Sprr1a* SesR-*H2BEGFP* was administered to *Krt8-Cre;Rosa-tdT* mice treated with either saline (B) or bleomycin (C). In saline-treated lungs, Krt8 expression (tdT+) was largely confined to the airway epithelium, with minimal EGFP signal (B). In contrast, bleomycin-treated lungs showed prominent co-localization of tdT+ and EGFP+ signals within fibrotic alveolar regions, suggesting targeted labeling of Krt8+ ADI cells *in vivo*. Scale bar = 20 μm.

Figure 4: **Transcriptomic convergence between EGFP-labeled *Sprr1a*+ cells and Krt8$^+$ ADI-like cells**. **A**, Violin plots showing the expression levels of structural (Krt8, Krt18, Krt7, Krt19) and injury-response (Cryab, Areg, Hbegf, Tnfrsf12a, Itgb6, Cldn18, Cldn4, Cdkn1a) genes in EGFP+ and EGFP- cells. These Krt8+ ADI-associated markers were consistently enriched in the EGFP+ population, indicating transcriptional alignment with the transitional alveolar epithelial cell state; **B**, Correlation heatmap illustrating transcriptomic similarity between EGFP+ and Krt8+ ADI-like cells based on the top 50 differentially expressed genes. Strong correlations were observed across Pearson (0.64), Spearman (0.81), and cosine similarity (0.76) metrics, supporting a shared gene expression profile between the two cell populations; **C**, UMAP projections of single-cell transcriptomes showing unsupervised Leiden clustering (left), distribution of EGFP+ cells (middle), and Krt8+ ADI-like cells (right). EGFP+ cells were predominantly localized within Krt8+ ADI-enriched clusters, although at lower density—likely due to incomplete viral transduction and/or limited EGFP transcript capture during scRNA-seq.

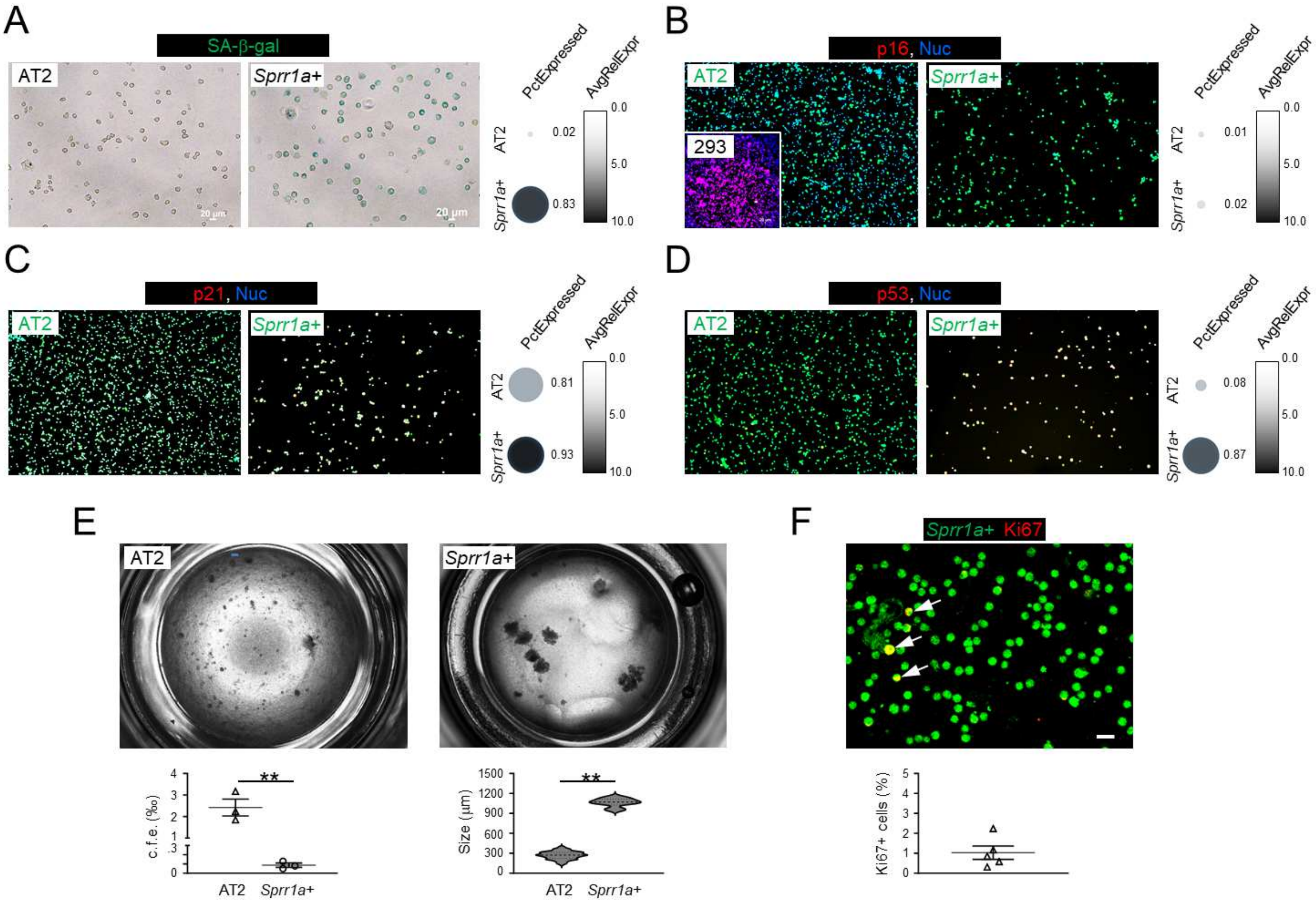

Figure 5: **Phenotypic characterization of uncultured *Sprr1a*+ cells isolated from mouse lungs**. **A**-**D**, Primary AT2 cells and *Sprr1a*+ cells were isolated from saline- or bleomycin-treated mouse lungs following transduction with AAV6.2FF vectors encoding either *Sftpc* sesRNA-*EGFP* or *Sprr1a* sesRNA-*EGFP*. Freshly sorted, uncultured cells were assessed for SA-β-gal activity (A) and expression of p16 (B), p21 (C), and p53 (D) by immunofluorescence staining. For each marker, the percentage of positive cells (PctExpressed) and the average relative expression (AvgRelExpr), representing the mean signal intensity normalized across positive cells, were quantified from at least three randomly selected microscopic fields; **E**, The organoid-forming capacity of AT2 cells and *Sprr1a*+ cells was evaluated in 3D Matrigel cultures. Colony formation efficiency (c.f.e.) ant organoid size were quantified across three independent experiments. Data are presented as mean ± SEM, $**p < 0.01$; **F**, Proliferative activity of EGFP-labeled *Sprr1a*+ cells were assessed and quantified following Ki67 (red) immunostaining. Results are presented as mean ± SEM from five randomly selected microscopic fields.

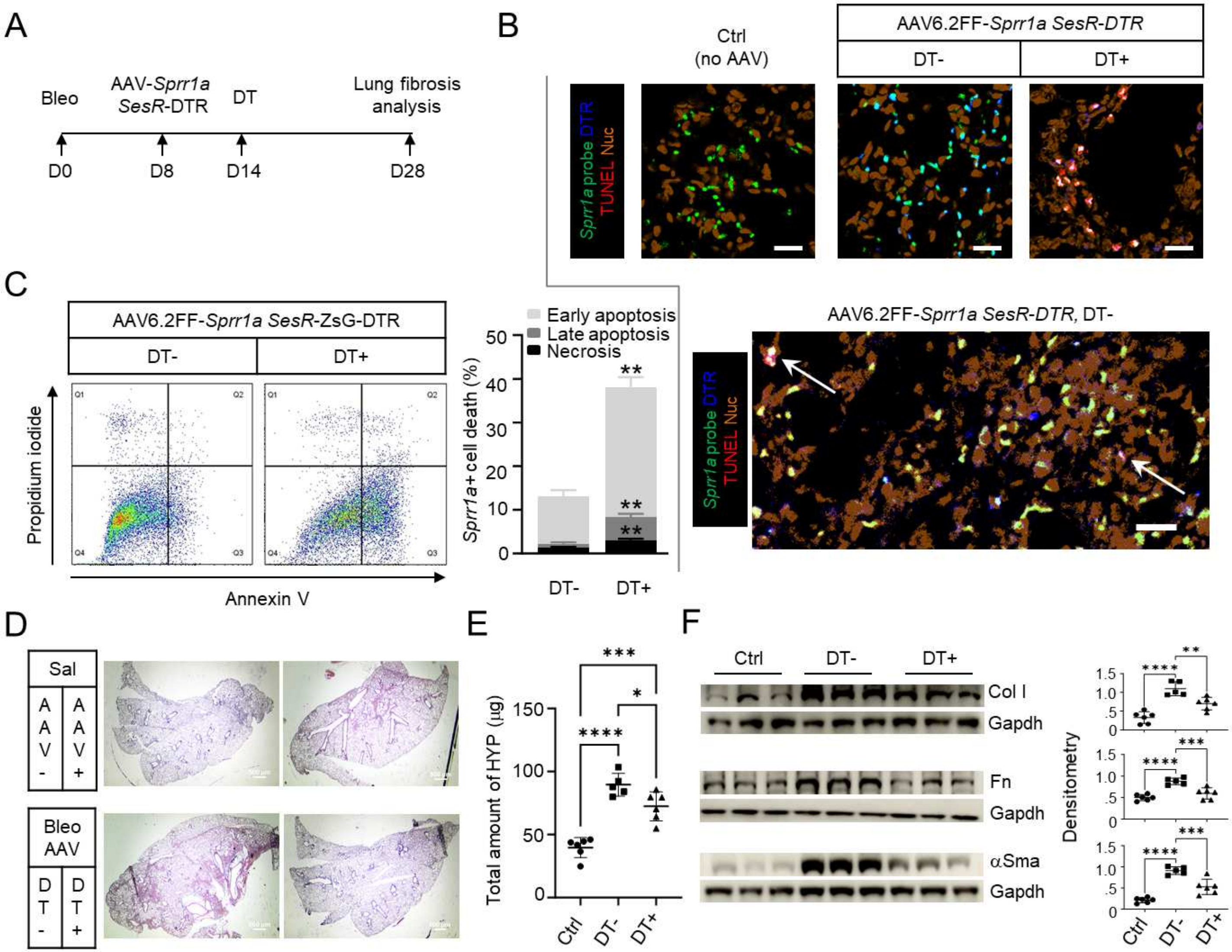

Figure 6: ***Sprr1a*-guided depletion of Krt8+ ADI cells attenuates pulmonary fibrosis in mice**. **A**, Schematic of the experimental design; **B**, Confocal images showing *Sprr1a*+ cell death at baseline and following DT treatment on Day 14 after bleomycin administration. *Sprr1a* mRNA was detected by RNA FISH (green), DTR protein by immunofluorescence (blue), and apoptosis by TUNEL (red); **C**, Quantification of *Sprr1a*+ cell death 24 hours after DT treatment using Annexin V and Propidium Iodide staining followed by flow cytometry; **D**, Masson's trichrome staining of lung tissues from bleomycin-treated mice infected with or without AAV6.2FF-*Sprr1a* sesRNA-*DTR*; **E**, Hydroxyproline content in lung tissues as a measure of collagen deposition; **F**, Protein levels of procollagen I (Col I), fibronectin (Fn), and α smooth muscle actin (αSma) in lung lysates from the same mice as described in D. Data are presented as mean ± SEM from 5-6 mice per group. $*p < 0.05$, $**p < 0.01$, $***p < 0.001$, $****p < 0.0001$. Scale bar = 20 μm.

Figure 2: ***SPRR1A* is a distinct transcriptomic marker of Krt8+ ADI cells in mice and aberrant basaloid cells in human IPF**. **A**, Analysis of *Krt8* and *Sprr1a* expression across various major mouse lung cell populations using a publicly available scRNA-seq dataset (GSE141259); **B**, Co-localization of *Sprr1a* mRNA with Krt8 and Sftpc proteins in lung tissues from bleomycin (bleo)-treated and saline (sal)-treated C57BL/6 mice by RNA FISH combined with immunofluorescence staining. **C**, Co-localization of SPRR1A, KRT17, and KRT5 in lung tissues from patients with IPF and healthy control subjects by immunofluorescence staining. Scale bar = 20 µm.

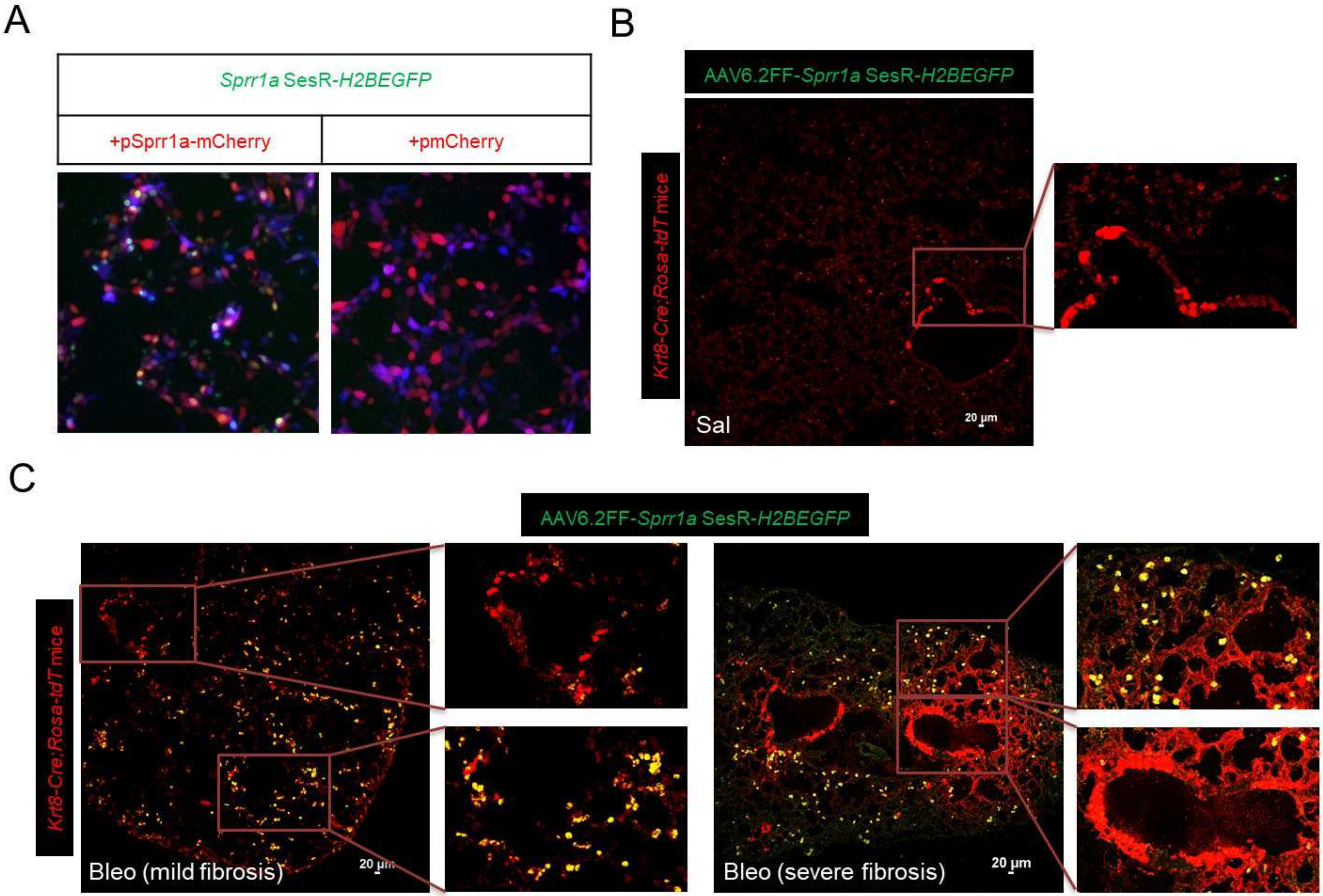

Figure 3: **Targeted *in vivo* labeling of Krt8+ ADI cells via *Sprr1a* mRNA-guided EGFP expression**. **A**, HEK 293 cells were co-transfected with *Sprr1a* SesR-*H2BEFGP* and either pSprr1a-mCherry or the control vector pmCherry. Confocal imaging showed EGFP expression selectively in cells co-expressing Sprr1a-mCherry (yellow), confirming *Sprr1a*-specific activation of the RNA-sensing system. **B** and **C**, AAV6.2FF-*Sprr1a* SesR-*H2BEGFP* was administered to *Krt8-Cre;Rosa-tdT* mice treated with either saline (B) or bleomycin (C). In saline-treated lungs, Krt8 expression (tdT+) was largely confined to the airway epithelium, with minimal EGFP signal (B). In contrast, bleomycin-treated lungs showed prominent co-localization of tdT+ and EGFP+ signals within fibrotic alveolar regions, suggesting targeted labeling of Krt8+ ADI cells *in vivo*. Scale bar = 20 µm.

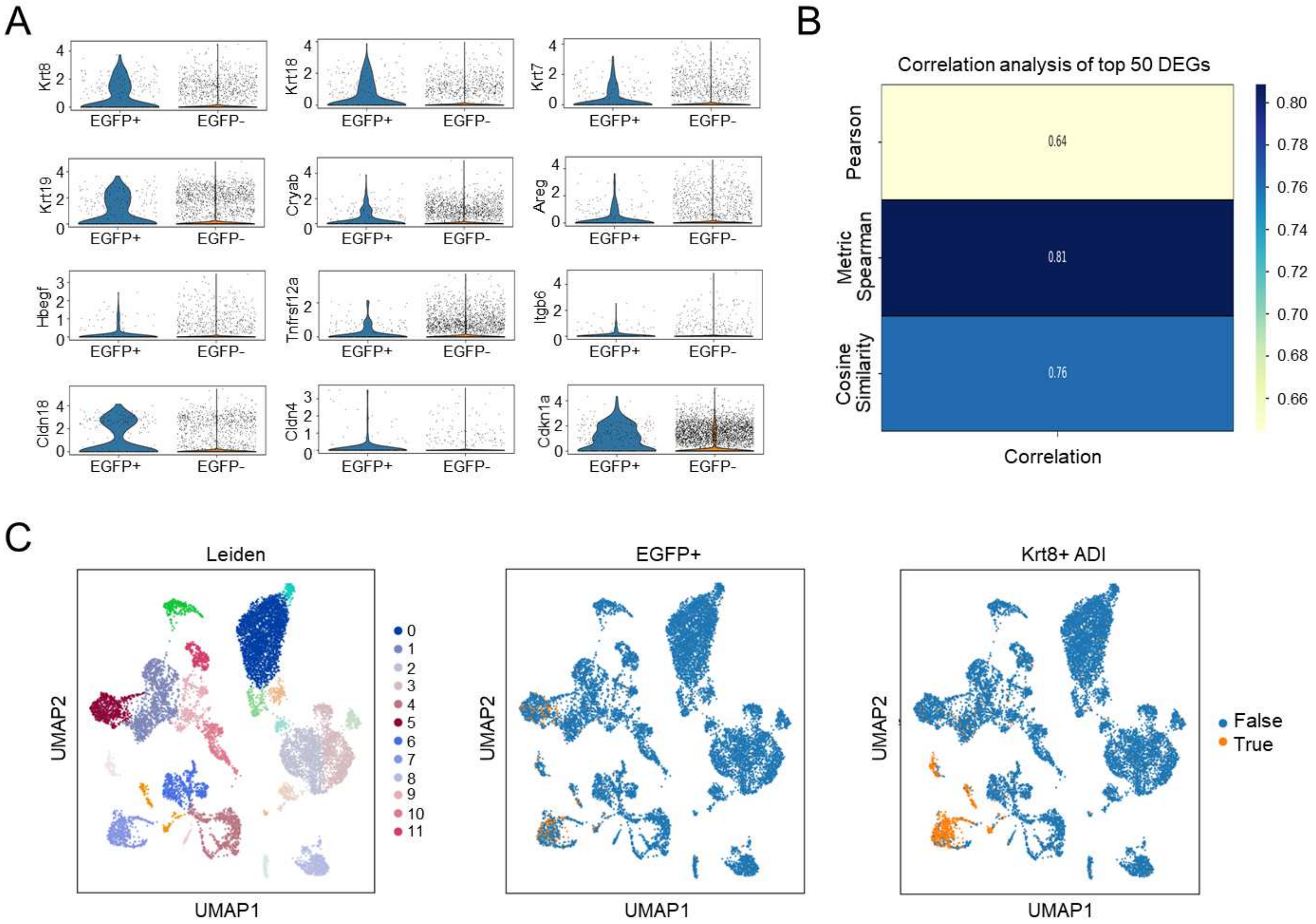

Figure 4: **Transcriptomic convergence between EGFP-labeled *Sprr1a*+ cells and Krt8$^{+}$ ADI-like cells**. **A**, Violin plots showing the expression levels of structural (Krt8, Krt18, Krt7, Krt19) and injury-response (Cryab, Areg, Hbegf, Tnfrsf12a, Itgb6, Cldn18, Cldn4, Cdkn1a) genes in EGFP+ and EGFP- cells. These Krt8+ ADI-associated markers were consistently enriched in the EGFP+ population, indicating transcriptional alignment with the transitional alveolar epithelial cell state; **B**, Correlation heatmap illustrating transcriptomic similarity between EGFP+ and Krt8+ ADI-like cells based on the top 50 differentially expressed genes. Strong correlations were observed across Pearson (0.64), Spearman (0.81), and cosine similarity (0.76) metrics, supporting a shared gene expression profile between the two cell populations; **C**, UMAP projections of single-cell transcriptomes showing unsupervised Leiden clustering (left), distribution of EGFP+ cells (middle), and Krt8+ ADI-like cells (right). EGFP+ cells were predominantly localized within Krt8+ ADI-enriched clusters, although at lower density—likely due to incomplete viral transduction and/or limited EGFP transcript capture during scRNA-seq.

Figure 6: ***Sprr1a*-guided depletion of Krt8+ ADI cells attenuates pulmonary fibrosis in mice**. **A**, Schematic of the experimental design; **B**, Confocal images showing *Sprr1a*+ cell death at baseline and following DT treatment on Day 14 after bleomycin administration. *Sprr1a* mRNA was detected by RNA FISH (green), DTR protein by immunofluorescence (blue), and apoptosis by TUNEL (red); **C**, Quantification of *Sprr1a*+ cell death 24 hours after DT treatment using Annexin V and Propidium Iodide staining followed by flow cytometry; **D**, Masson's trichrome staining of lung tissues from bleomycin-treated mice infected with or without AAV6.2FF-*Sprr1a* sesRNA-*DTR*; **E**, Hydroxyproline content in lung tissues as a measure of collagen deposition; **F**, Protein levels of procollagen I (Col I), fibronectin (Fn), and a smooth muscle actin (aSma) in lung lysates from the same mice as described in D. Data are presented as mean ± SEM from 5-6 mice per group. *$p < 0.05$, **$p < 0.01$, ***$p < 0.001$, ****$p < 0.0001$. Scale bar = 20 µm.

## Supplementary Files

This is a list of supplementary files associated with this preprint. Click to download.

- SupplementalInfo.pdf
